# Outbreak of Porcine Epidemic Diarrhea in Suckling Piglets, China

**DOI:** 10.3201/eid1801.111259

**Published:** 2012-01

**Authors:** Rui-Qin Sun, Ru-Jian Cai, Ya-Qiang Chen, Peng-Shuai Liang, De-Kun Chen, Chang-Xu Song

**Affiliations:** Guangdong Academy of Agricultural Sciences Veterinary Medical Institute, Guangzhou, China (R.-Q. Sun, R.-J. Cai, C.-X. Song);; Northwest A & F University, Xi’an, Shanxi, China (R.-Q. Sun, D.-K. Chen);; Inner Mongolia Agriculture University, Huhhot, Inner Mongolia, China (Y.-Q. Chen);; Henan Agriculture University, Zhengzhou, Henan, China (P.-S. Liang)

**Keywords:** porcine epidemic diarrhea, sow milk, transmission route, suckling piglets, China

**To the Editor:** Beginning in October 2010, porcine epidemic diarrhea (PED), caused by a coronaviral infection affecting pigs, emerged in China in an outbreak characterized by high mortality rates among suckling piglets. The outbreak overwhelmed >10 provinces in southern China, and >1,000,000 piglets died. This outbreak was distinguished by ≈100% illness among piglets after birth (predominantly within 7 days and sometimes within only a few hours) and death rates of 80%–100% (Technical Appendix Table 1). Few sows or boars showed any clinical signs during the outbreak, which is not consistent with a recent report from Thailand (*1*). In that outbreak during late 2007, pigs of all ages were affected, exhibiting different degrees of diarrhea and no appetite. We characterized the genetic variation of the PED virus (PEDV) that caused a large-scale outbreak in China during 2010–2011 and compared it with viruses in other outbreaks. We also report a possible novel transmission pathway for PEDV.

A total of 177 samples (intestine, stool, and maternal milk) were collected from pigs from different farms who had diarrhea; 100% of farms had >1 porcine sample positive for PEDV. A total of 125/177 porcine samples were confirmed as positive for PEDV by reverse transcription PCR using primers as described (*2*). PEDV was detected in 105 (82.0%) of 128 fecal samples and 20 (40.8%) of 49 sow milk samples. Piglets infected with PEDV showed mild hemorrhage, undigested curdled milk in the stomach, and thin-walled intestines with severe mucosal atrophy and foamy fluid (data not shown).

The spike (S) gene of the family *Coronaviridae* has a high degree of variation and can induce neutralizing antibody (*3*). Reverse transcription PCR products of the 651-bp partial S gene of PEDV and the deduced amino acid sequences were aligned by using ClustalW (www.genome.jp/tools/clustalw), and a neighbor-joining tree with 1,000 bootstraps was constructed. Sequences of the S genes from this outbreak were 99.1%–100.0% homologous and had 88.7%–98.9% nt identity with all reference strains (Technical Appendix Table 2), 98.5%–98.9% with Thailand strains, and 94.5%–95.1% with vaccine strain CV777. The partial S gene deduced amino acid sequences were compared and also showed a high degree of homology (98.0%–100.0%); they had 85.3%–98.7% identity with all reference strains listed in Technical Appendix Table 2, 98.0%–98.7% with Thailand strains, and 93.3%–94.7% with vaccine strain CV777 (data not shown).

Phylogenetic analysis indicated that the PEDV in the China outbreak was different from foreign and other domestic strains on the basis of the reported partial S gene sequences. All new strains were clustered in the same branch, close to the cluster of Thailand strains, and far from the cluster of vaccine strain CV777 (Figure).

**Figure:**
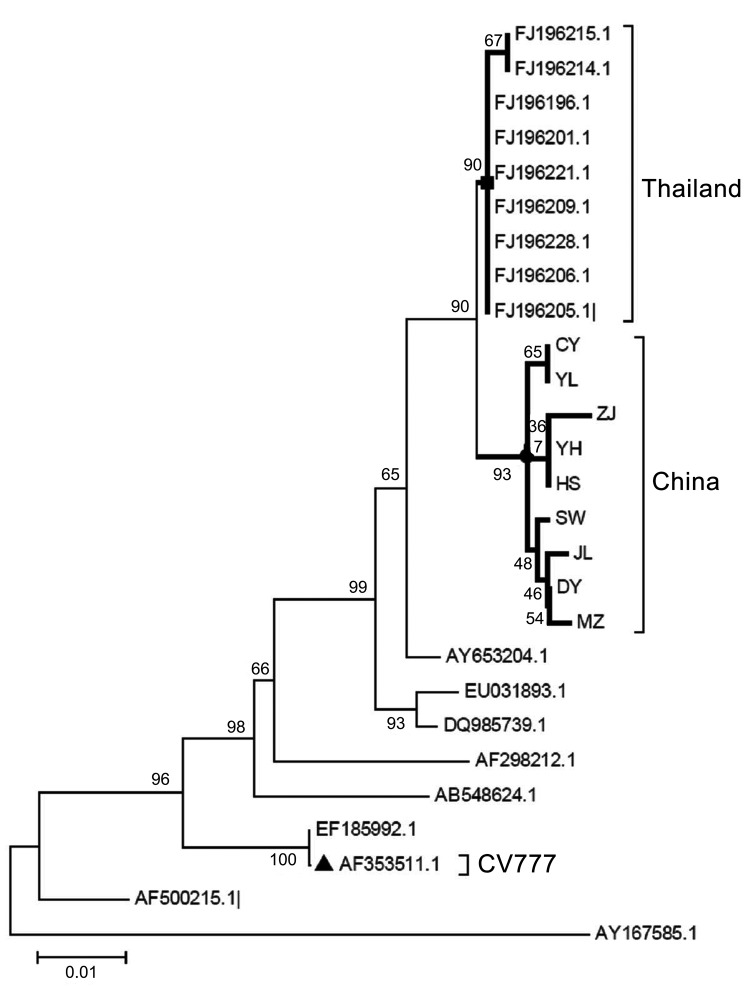
Phylogenetic tree constructed by using the neighbor-joining method based on the 9 porcine epidemic diarrhea virus (PEDV) sequences identified in a study of porcine epidemic diarrhea in China. Partially amplified spike genes of the PEDV isolates plus 18 PEDV sequences downloaded from GenBank were compared. Sequences included in each cluster are listed in Technical Appendix Table 3. Strains from Thailand and China and the CV777 vaccine strain are indicated. Scale bar indicates nucleotide substitutions per site.

In the China outbreak, PEDV caused severe diarrheal disease in piglets; heavy economic losses in many provinces resulted, despite use of commercial vaccines (inactivated transmissible gastroenteritis [TGEV H] and porcine epidemic diarrhea [CV777]). To determine why the vaccines showed poor efficacy, we investigated evolution of the virus. Comparison of amino acid sequences from isolates from the outbreak and from the CV777 vaccine strain showed 9 amino acid mutations of fragments containing major hydrophilic regions: 16 (L→H), 18 (S→G), 22 (V→I), 44 (T→S), 89 (G→S), 100 (A→E), 107 (L→F), 130 (I→V) and 160 (I→F) (Technical Appendix Figure, panel A). Three of these 9 mutations were at positions 16, 18, and 22 in the isolates from China; they influenced the hydrophobicity of the S protein as compared with that for CV777 (Technical Appendix Figure, panel B).

Phylogenic analysis showed that strain CV777 did not cluster with current common strains and showed considerable genetic distance from them. Isolates in the outbreak in China had only a minor nucleotide sequence variation from the Thailand isolates, indicating that the virus has a high genetic relatedness to the Southeast Asia strain. However, previous studies showed that isolates from Europe, South Korea, and China were serologically identical to the prototype CV777 strain (*1**,**4*).

To our knowledge, fecal–oral transmission is probably the main or only route of PEDV transmission (*5**–**7*). In our study, if a fecal sample from a sick piglet was found to be positive for PEDV, we also collected and studied milk from its mother. These results showed that PEDV was present in sow milk (Technical Appendix Table 3), but the detection rate was lower for these samples (40.8%) than for the fecal samples (82.0%).

On the basis of these results, we hypothesize that sow milk could represent a possible (and potentially major) route for the vertical transmission of PEDV from sow to suckling piglet. This hypothesis could be indirectly verified by our field observation that piglet death rates decreased as a result of fostering (data not shown). Our findings show that PEDV was identified not only in fecal samples from sick piglets, as expected, but also in the milk of the sow, which suggests vertical transmission of the virus.

## Supplementary Material

Technical Appendix Current farms status in this study, China.
