# Supplementary material for: Outbreak of Porcine Epidemic Diarrhea in Suckling Piglets, China
Source: Emerg Infect Dis. 2012 Jan;18(1):161–3. doi: 10.3201/eid1801.111259 (PMC3381683; doi:10.3201/eid1801.111259)
Supplement: Technical Appendix — Current farms status in this study, China. [file 11-1259-Techapp_2p.pdf]

Article DOI: http://dx.doi.org/10.3201/eid1801.111259

Outbreak of Porcine Epidemic Diarrhea in Suckling Piglets, China

Technical Appendix

Table 1. Current farms status in this study, China

Farm	No. sows	Vaccination*	Illness rate ,%/y	Mortality rate. %
YL	1,000	Yes	90	50
HS	1,400	No	100	80
CY	300	Yes	80	90
SW	1,000	Yes	80	100
DY	2,000	Yes	95	100
JL	80	No	75	80
ZJ	90	No	100	90
MZ	100	No	60	100

*Sows were vaccinated with divalent inactivated transmissible gastroenteritis (TGE) and porcine epidemic diarrhea (PED) vaccine before delivery.

Table 2. Accession numbers, district, and collection date of the sequences used*

Accession no.	District	Strain	Collection date
–	Shen-Zhen, Guangdong	JL	2010 Nov
–	Da-Yang, Guangdong	DY	2010 Dec
–	Qing-Yuan, Guangdong	YH	2011 Feb
–	Qing-Yuan, Guangdong	YL	2011 Mar
–	Shi-Wan, Guangdong	SW	2011 Apr
–	Bo-Luo, Guangdong	CY	2011 May
–	He-Shan, Guangdong	HS	2011 May
–	Mei-Zhou, Guangdong	MZ	2011 Jun
	Zhan-Jiang, Guangdong	ZJ	2011 Jul
FJ196196.1	Nakornpathom (Thailand)	07NP01	2007 Dec
FJ196201.1	Chonburi (Thailand)	08CB05	2008 Mar
FJ196205.1	Nakornpathom (Thailand)	08NP03	2008 Jan
FJ196206.1	Nakornpathom (Thailand)	08NP04	2008 Jan
FJ196209.1	Nakornpathom (Thailand)	08NP07	2008 Mar
FJ196214.1	Ratchaburi (Thailand)	08RB02	2008 Jan
FJ196215.1	Ratchaburi (Thailand)	08RB03	2008 Jan
FJ196221.1	Chonburi (Thailand)	KU01CB08	2008 Mar
FJ196228.1	Ratchaburi (Thailand)	KU08RB08	2008 Mar
AY653204.1	Nan-Jing (China)	JS-2004-2	2004 Jul
EU031893.1	Gan-Su (China)	DX	2007 Aug
AF298212.1	South Korea	–	2002 Aug
AF500215.1	South Korea	–	2002 Apr
AY167585.1	South Korea	Chinju99	2002 Oct
EF185992.1	Lan-Zhou (China)	LZC	2006 Dec
AB548624.1	Japan	MK	2010 Mar
DQ985739.1	Hei-Long-Jiang (China)	LJB/03	2006 Sep
AF353511.1	China	CV777	2001 Feb

*–, sequences obtained from present outbreak have no accession no.

Table 3. Detection of PEDV-affected piglets and sows samples by RT-PCR, China*

Farm	Feces and intestine (no. positive/total)	Milk (no. positive/total)
YL	10/10	4/10
HS	24/24	4/6
CY	8/14	2/6
SW	4/6	1/5
DY	26/30	5/12
JL	3/6	–
ZJ	16/22	–
MZ	4/6	–

*PEDV, porcine epidemic diarrhea virus; –, no corresponding samples.

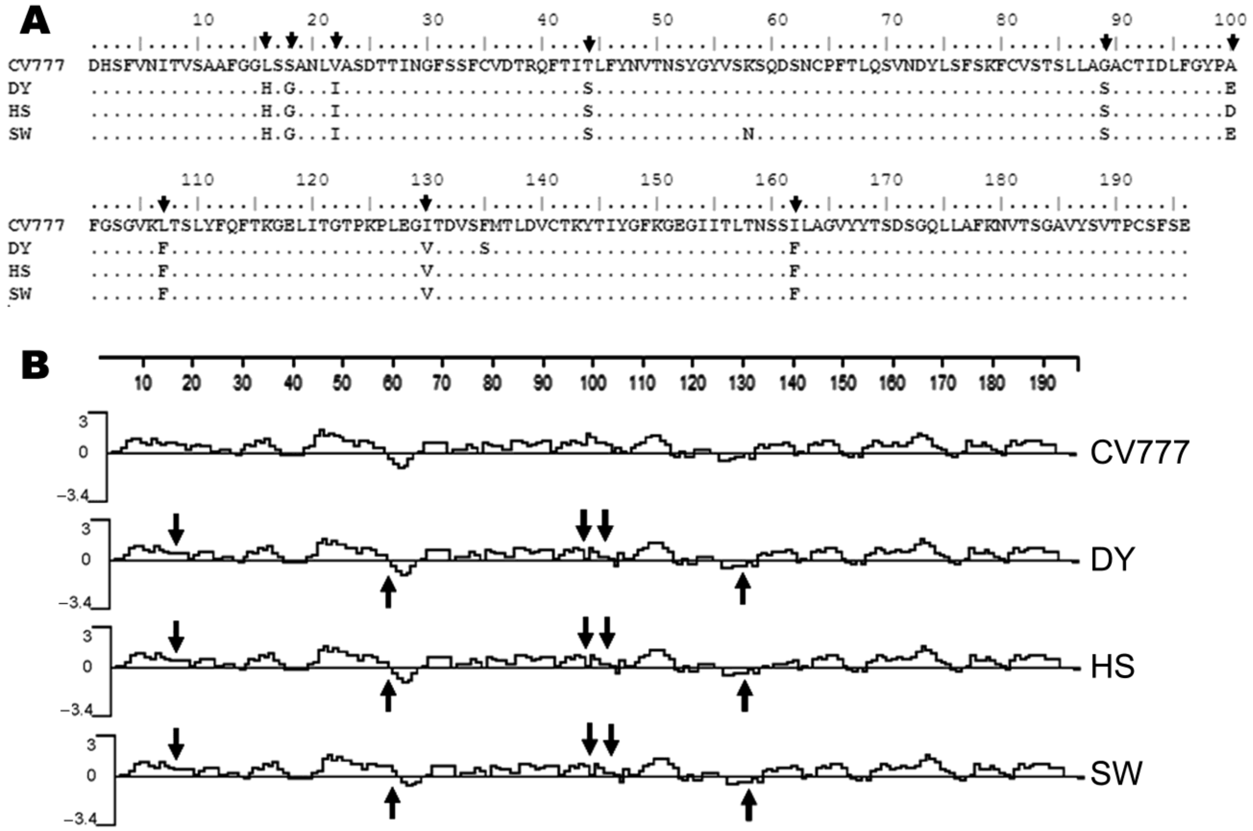

Figure. A) Deduced amino acid sequence comparison of the spike (S) proteins between these isolates from China (DY, HS and SW) and the reference CV777 strain. Only the amino acids different from those in the consensus sequence are indicated. B) Hydrophobicity plots of S generated by the Kyte and Doolittle method by using DNAstar program (www.dnastar.com). Major areas of difference are indicated by arrows.
